# Production of Enantiopure Chiral Epoxides with *E. coli* Expressing Styrene Monooxygenase

**DOI:** 10.3390/molecules26061514

**Published:** 2021-03-10

**Authors:** Dominika Gyuranová, Radka Štadániová, Zuzana Hegyi, Róbert Fischer, Martin Rebroš

**Affiliations:** 1 Faculty of Chemical and Food Technology, Institute of Biotechnology, Slovak University of Technology in Bratislava, Radlinského 9, 812 37 Bratislava, Slovakia; dominika.gyuranova@stuba.sk (D.G.); zuzana.hegyi@stuba.sk (Z.H.); 2 Faculty of Chemical and Food Technology, Institute of Organic Chemistry, Catalysis and Petrochemistry, Slovak University of Technology in Bratislava, Radlinského 9, 812 37 Bratislava, Slovakia; radka.stadaniova@stuba.sk (R.Š.); robert.fischer@stuba.sk (R.F.)

**Keywords:** styrene monooxygenase, whole-cell biocatalysis, chiral epoxides

## Abstract

Styrene monooxygenases are a group of highly selective enzymes able to catalyse the epoxidation of alkenes to corresponding chiral epoxides in excellent enantiopurity. Chiral compounds containing oxirane ring or products of their hydrolysis represent key building blocks and precursors in organic synthesis in the pharmaceutical industry, and many of them are produced on an industrial scale. Two-component recombinant styrene monooxygenase (SMO) from *Marinobacterium litorale* was expressed as a fused protein (StyAL2StyB) in *Escherichia coli* BL21(DE3). By high cell density fermentation, 35 g_DCW_/L of biomass with overexpressed SMO was produced. SMO exhibited excellent stability, broad substrate specificity, and enantioselectivity, as it remained active for months and converted a group of alkenes to corresponding chiral epoxides in high enantiomeric excess (˃95–99% ee). Optically pure (*S*)-4-chlorostyrene oxide, (*S*)-allylbenzene oxide, (2*R*,5*R*)-1,2:5,6-diepoxyhexane, 2-(3-bromopropyl)oxirane, and (*S*)-4-(oxiran-2-yl)butan-1-ol were prepared by whole-cell SMO.

## 1. Introduction

Styrene monooxygenase (SMO) is a flavoprotein monooxygenase that incorporates a single oxygen atom into a styrene molecule to form styrene oxide [1]. Originally found in *Pseudomonas* and *Rhodococcus* species, SMO is comprised of two subunits—FAD-dependent monooxygenase (StyA) and NADH-dependent flavin-reductase (StyB)—encoded by *styA* and *styB* of *styABDE* gene cluster responsible for the degradation of styrene [2,3]. Since the epoxidation of styrene to styrene oxide by SMO is highly enantioselective, *styA* and *styB* are possible candidates for biocatalysis. Initially, wild-type SMOs of *Pseudomonas* were widely employed for biotransformation of styrene, but, to date, many other bacterial species that share styrene degradation pathways have been described [1]. Such discoveries, together with the expansion of recombinant technologies, metagenomics, and protein engineering, have led to the generation of SMOs with higher stability, selectivity, and activity towards a broad substrate spectrum, usually expressed as recombinant enzymes in *Escherichia coli* [2,4,5,6,7]. Significant progress was achieved by Panke et al. in previous studies [8,9,10,11]. In 1998, they characterised the styrene degradation metabolic pathways in *Pseudomonas* sp. and, in 2002, produced 388 g of enantiopure (*S*)-styrene oxide in a 30 L scale by the whole-cell SMO expressed in *E. coli* JM101, which is the highest (*S*)-styrene oxide yield achieved so far. However, only a low cell concentration (12 g/L) was reached during fermentation, despite being operated on a pilot-scale [11]. Although recombinant production of SMO has been known for several decades, the majority of biotransformations are performed using whole-cell SMO that may cause a decline in enantiomeric excess (ee) of chiral products because of possible side reactions [2,12,13,14]. Few attempts of SMO isolation have been reported, but only the small-scale expression of StyA and StyB separately was successful [4,15,16].

Chiral epoxides comprise a group of important building blocks and intermediates of biologically active pharmaceuticals [17]. To date, many epoxides have been applied to the synthesis of various drugs, for instance, antiviral agents (Indinavir, Cidofovir), chemotherapeutics (Paclitaxel, Docetaxel), antidepressants (Tomoxetine, Fluoxetine), or sleeping disorder treatments (Tesamelteon) [2,17,18]. Among them, styrene oxide, glycidol, epichlorohydrin, chlorostyrene oxide, and others are produced on a large scale [17]. However, biocatalysis still suffers from the low availability of enzymes in large quantities and is often replaced by chemical catalysis on an industrial scale. Sharpless and Jacobsen oxidation that demand extreme reaction conditions and suffer from poor enantioselectivity are mostly employed in pilot-scale chiral epoxides preparation [14,17]. Further improvement of SMO production (e.g., fermentation optimisation, enzyme stability improvement, effective purification development) significantly improves the production cost and enantiopurity of chiral epoxides. Thanks to their broad substrate scope, further application of SMOs on an industrial scale could also extend the spectrum of optically active chiral compounds produced by green chemistry.

In this work, the gene of SMO from *Marinobacterium litorale* was cloned to a pET28b(+) vector and expressed in *Escherichia coli* BL21(DE3) under the control of isopropyl-β-D-thiogalactopyranoside (IPTG) inducible *lac*T7 promoter. Recombinant SMO originating from *M*. *litorale* has been expressed and characterised in [2]. However, the substrate specificity of the enzyme was evaluated only towards few structurally similar styrene derivatives. Used SMO encoded by *styA* and *styB* was fused by the linker L2 designed in [16] (StyAL2StyB). Firstly, induction conditions were optimised in the flask, then the production of SMO was upscaled to a 1.5 L scale for high cell density production. SMO was further purified by affinity chromatography and characterised. The affinity of SMO towards 34 various structurally different alkenes was tested. Selected biotransformations were upscaled for the production of optically pure chiral epoxides.

## 2. Results and Discussion

### 2.1. Expression of SMO

SMO expression was optimised by varying media composition, IPTG concentration, and induction temperature. When cells reached a concentration above 0.6 g/L during cultivation in LB medium, the formation of a blue pigment was observed. This phenomenon was described before and is the result of tryptophanase coexpression, which together with SMO transform tryptophan to indigo in cultivation media [19]. In *E. coli*, excess tryptophan induces the expression of tryptophanase that hydrolyses tryptophan to indole. The primary function of indole is to regulate cellular processes [20], but its structural similarity to styrene also makes it a substrate of SMO that converts indole to indoxyl. Further isomerisation of indoxyl then generates indigo. Production of indigoid dyes is a widely applied high-throughput screening method for monooxygenase directed evolution library screenings [3,21]. In the present work, however, the presence of indigo was also observed in noninduced cultures grown on LB agar plates, demonstrating only unregulated enzyme expression (data not shown). Therefore, mineral–amino acids free M9 medium was applied for SMO expression. Due to the lack of nutrients, the cell growth in M9 medium was slower; however, comparable cell concentrations and SMO specific activity were reached (Figure S1). SDS-PAGE protein profiles confirmed the expression of recombinant SMO with a molecular size of 66 kDa, consisting of StyA (45 kDa) and StyB (19 kDa) [2], which corresponds to the predicted size of the enzyme.

Different concentrations of IPTG resulted in the same SMO expression, as identified by SDS-PAGE (Figure S2). However, even at IPTG concentrations lower than 1 mM, a high amount of SMO was formed in inclusion bodies, probably due to the high induction temperature [3]. Its decrease to 20 °C led not only to an enhancement of SMO solubility (Figure S3), but also to a 70% increase in SMO specific activity (Figure S1).

The final optimised flask conditions in M9 medium were 0.25 mM IPTG and 20 °C induction for 22 h, where a specific activity of 12 U/g_DCW_ of SMO was achieved.

### 2.2. High Cell Density Fermentation

High cell density (HCD) fermentation is a well-established method for recombinant enzyme production. However, the process performance and productivity highly depend on many factors (fermentation media composition, feeding strategy, induction conditions, expression host, gene of the desired product) and require optimisation [22,23,24].

**Figure 1:**
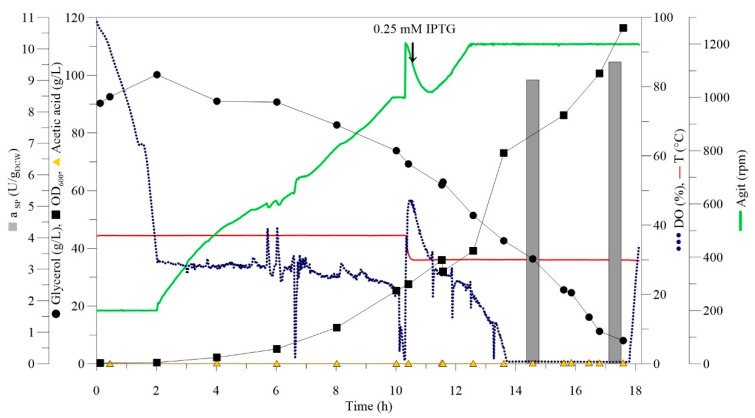
HCD batch fermentation of *E. coli* expressing SMO performed on the 1.5 L scale.

To upscale the SMO production, 0.5 L HCD fermentations were performed with glycerol as a carbon source. As the utilisation of glycerol by *E. coli* generates organic acids such as acetate, which significantly decreases biomass growth at a concentration above 2 g/L [22], fermentation was performed with a fixed pH strategy. The expression of SMO was initially induced at a low cell concentration of 0.4 g_DCW_/L, but under these conditions, the cell growth was inhibited immediately. The concentration of IPTG is one of the crucial factors that affect the growth of *E. coli*, the level of expression, and also the activity of a recombinant enzyme [22,24,25]. As previous studies showed that excessive IPTG concentration could inhibit or even repress biomass growth [22,23], we assume that the concentration of 0.4 g_DCW_/L was too low for the induction. Expression of SMO was further induced at 15 g_DCW_/L (OD_600_ = 50) for 5.5 h (Figure S4), where a rising trend of SMO activity was demonstrated. After 16 h of fermentation, and complete glycerol utilisation, 31 g_DCW_/L of biomass and 10.5 U/g_DCW_ of SMO were achieved. HCD fermentation was then upscaled to 1.5 L (Figure 1, Figure S5). The results are summarised in Table 1.

The final, optimised HCD fermentation protocol for *E. coli* BL21(DE3) expressing recombinant SMO was as follows: M9 inoculation medium and induction with 0.25 mM IPTG at 30 °C and 15 g_DCW_/L in a batch mode. Following the protocol, HCD fermentation with 1.5 L provided 35 g_DCW_/L of overexpressed SMO with a specific activity of 9.6 U/g_DCW_.

### 2.3. Isolation and Characterisation of Recombinant SMO

#### 2.3.1. Purification of Recombinant SMO

Due to the presence of the His tag, recombinant SMO was isolated by immobilised Ni^2+^ affinity chromatography according to [26] (Figure S6). Initially, the purification was optimised on a small-scale, where only a small amount of SMO (Table S1) was recovered per single run. Upscaled purification achieved a 4-fold higher SMO yield (Table S1), but in general, only up to 0.2 mg of purified SMO per mL of crude extract were isolated (approximately 0.01 g_SMO_ per g_DCW_). Since this is not the first report of SMO isolation causing an obstacle [21], the low purification yield and substantial decrease in the specific activity of purified SMO compared to its crude form (discussed below) are probably caused by the loss of FAD during purification [27].

#### 2.3.2. Determination of Optimal pH, Temperature, and Storage Conditions

**Figure 2:**
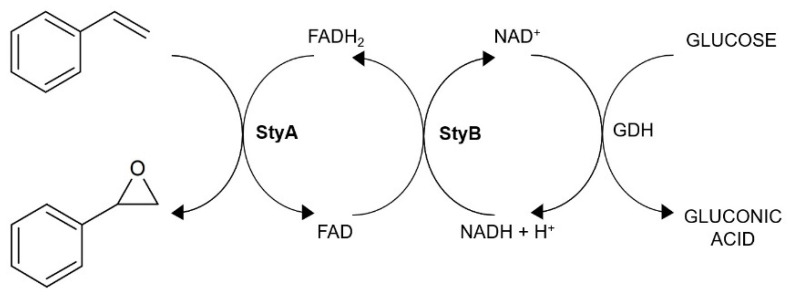
Biocatalytic cascade of styrene epoxidation involving GDH cofactor regeneration system.

As Figure 2 shows, the mechanism of styrene epoxidation by SMO is quite complex and, due to the StyB dependence on reduced NADH, also requires a cofactor regeneration system in a cell-free system. Therefore, a glucose dehydrogenase (GDH) regeneration system was employed [26].

**Figure 3:**
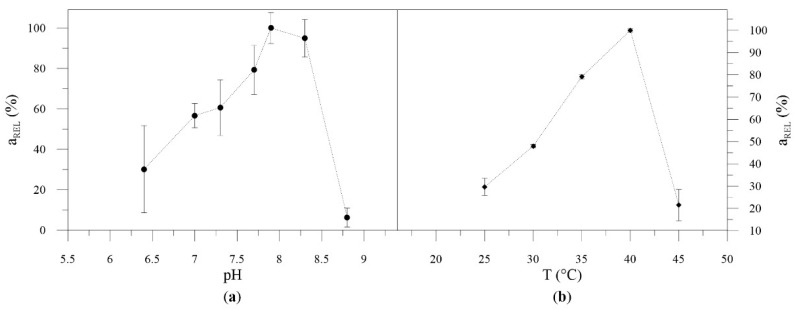
pH (**a**) and temperature (**b**) profile of purified SMO.

SMO exhibited a maximum activity at pH 7.8, but was highly active at the range 7.7–8.3 (Figure 3a). Since gluconic acid was formed in the reaction (Figure 2), a pH of 8 was applied similar to a previous report [2]. The SMO reached a maximum activity at 40 °C (Figure 3b). However, evaluation of specific activity during SMO isolation revealed that, with increasing purity of enzyme, the catalytic activity decreases significantly (Table S2). Therefore, further application of whole cells and crude enzyme extract was applied, which slightly shifted the optimal temperature (Figure S7). In its crude form, SMO retained 70% of its initial activity after 16 months of storage at −80 °C (Figure S8). Moreover, whole cells with SMO were also suitable for repeated biotransformations (Figure S9).

### 2.4. Biotransformation of Alkenes

The substrate specificity of SMOs has been investigated for many years [12,13,28,29]. Initially, SMOs were considered as enzymes with a narrow substrate spectrum, especially those originating from *Pseudomonas* and *Rhodococcus* species. However, recent studies described novel SMOs from various bacteria or metagenome, which provided novel insights into the catalytic mechanism of styrene epoxidation and, thereby, enabled the generation of SMOs with improved biocatalytic activities and a broader substrate spectrum [4,6,14,30]. So far, all studies have confirmed that few structural characteristics of the substrate significantly impact the biocatalysis: acyclic double bonds are preferred; an electron-withdrawing group conjugated to a double bond, α-/β-substitution of styrene/styrene derivatives, as well as 2-substitution of an aromatic ring decrease activity [5,6,12,13,31].

The affinity of SMO towards a wide range of alkenes (Table 2) was examined. Screened substrates were divided into the following groups: styrene derivatives (**1**), unconjugated aromatic alkenes (**2**), unsaturated aldehydes and ketones (**3**), unsaturated carboxylic acid and esters (**4**), linear alkenes (**5**), and unsaturated amines and imines (**6**–**8**).

The substitution of a styrene derivativesˈ double bond seemed to be unfavourable for epoxidation, as none of the **1b**–**e** alkenes were converted to epoxide. Both double bond and aromatic ring substitution presumably acted as a steric barrier to the active site of the enzyme. On the other hand, the bioconversion of 4-Cl-substituted styrene derivative (**1f**) and unconjugated allylbenzene (**2a**) was high. In both cases, a higher activity of SMO compared to styrene was achieved. Both 4-chlorostyrene oxide and allylbenzene oxide were formed in the upscaled process, with excellent conversion (more than 99%), ee, and yield (Table 3).

2-hydroxyl substitution of the aromatic ring also seemed unfavourable (**2b**). SMO showed no affinity towards **5a**–**c**, **e**, and **f**, but was highly active in the epoxidation of **5d**, implying a trend of rising activity with increasing distance between the double bond and the hydroxyl group. A similar trend was confirmed compared to the epoxidation of bromo-alkenes **5i** and **5j**. On the contrary, the activity of SMO decreased with increasing distance of the oxirane ring from the double bond (**5g**, **5h**). Eventually, both epoxides were converted to diepoxides, but the conversion of longer **5h** and ee of the corresponding diepoxide was lower compared to **5g**.

From the group of unsaturated aldehydes and ketones, only **3b** was transformed to epoxide with excellent enantiopurity (ee ˃ 99%). Interestingly, **3b** only differs from **3a** by one methyl- substitution on C_2_ carbon, which supports the previous finding of SMO activity enhancement by double bond methylation [14]. On the other hand, no activity towards **4c** was observed, suggesting that the combination of methyl- and carbonyl- substituents was crucial. Unfortunately, SMO showed no affinity to any tested unsaturated ketone, carboxylic acid, ester, amine, or imine.

### 2.5. Upscale Production of Chiral Epoxides

To evaluate the scope of SMO applicability, upscaled biotransformations were performed. It was found that the crude extract of SMO was not suitable for upscale epoxidations, as many undesired compounds were repeatedly isolated from crude reaction mixtures besides epoxides (data not shown), since additional purification steps provided only trace final amounts of epoxides. Therefore, whole-cell SMO was employed, providing the same enantioselectivity and even higher conversion compared to crude extracts (Table 3). As Table 3 shows, almost all epoxides were produced in (*S*) configuration, similar to previous reports [28,29,32]. As far as we know, this is the first report of (2*R*,5*R*)-1,2:5,6-diepoxyhexane and 2-(3-bromopropyl)oxirane enzymatic production by SMO in such a configuration and/or enantiopurity.

#### Evaluation of Chiral Epoxides

The application of chiral epoxides prepared in this work has not been reported so far. However, structurally similar (*R*)-**1f** epoxide is a possible intermediate in the synthesis of neuroprotective agent Eliprodil. Similarly, (*R*)-3-**1f** epoxide serves as an intermediate of antidiabetic and antiobesity agents and is produced on an industrial scale (100–4000 L) [17,33]. 2-methyl-**2a** epoxide is an intermediate for the synthesis of (*R*)-(−)-mevalonolactone, a key intermediate of cellular biological processes and regulation [34]. The possible retrosynthetic pathway of these epoxides is shown in Scheme S1.

Despite the rising trend of biocatalysis or chemo-biocatalysis involvement in industrial production, chiral epoxides are still preferably produced by the manner of chemical catalysis. The ability of SMOs to operate at mild reaction conditions, together with their tremendous enantioselectivity, thereby emphasise their potential for industrial applications.

## 3. Materials and Methods

### 3.1. Chemicals and Media

Kanamycin was purchased from Gibco^®^ (Life Technologies, Glasgow, UK). NAD^+^ was purchased from Prozomix (Haltwhistle, UK). *N*-hexadecane was purchased from Alfa Aesar (Haverhill, MA, USA). Ethyl acetate was purchased from Merck (Darmstadt, Germany). Styrene, styrene oxide, and 4-chlorostyrene oxide were purchased from Sigma Aldrich (St. Louis, MO, USA). 4-chlorostyrene was purchased from Carbosynth (San Diego, CA, USA). Other alkenes and standards of epoxides were provided by the Department of Organic Chemistry of the Slovak University of Technology.

Lysogeny broth (LB) was prepared according to [35]. Semidefined medium contained 90 g/L glycerol, 10 g/L tryptone, 5 g/L (NH_4_)_2_SO_4_, 3.64 g/L NaH_2_PO_4_.2H_2_O, 4.53 g/L K_2_HPO_4_, 4 g/L citric acid, 1% (*v*/*v*) trace element solution, 1 g/L MgSO_4_, 7H_2_O. Mineral M9 medium contained 5 g/L glycerol, 0.5 g/L MgSO_4_.7H_2_O, 0.11 g/L CaCl_2_, 0.01 g/L thiamine-HCl, and 5% (*v*/*v*) M9 salts (85 g/L Na_2_HPO_4_.12H_2_O, 15 g/L KH_2_PO_4_, 2.5 g/L NaCl, 5 g/L NH_4_Cl). All media were supplemented with kanamycin to a final concentration of 30 µg/mL.

### 3.2. Construction of Expression Vectors

Codon-optimised genes *styA* and *styB* [2] encoding SMO from *Marinobacterium litorale* were linked with linker WYHHHH according to [16] and were purchased from Generay Biotech Co., Ltd. (Shanghai, China). Genes were inserted into a pET28b vector system. Plasmids were then transformed into competent cells of *Escherichia coli* BL21(DE3).

### 3.3. Expression of SMO

A single colony of *E. coli* BL21(DE3) with pET28b(+)SMO was transferred to 3 mL of LB/M9 medium supplemented with kanamycin (30 µg/mL) and cultivated at 37 °C and 200 rpm overnight. A total of 100 mL of LB/M9 medium in a 500 mL shaking flask were inoculated with 1% (*v*/*v*) of overnight culture and cultivated at 37 °C and 200 rpm until OD_600_ reached 0.5–0.7. The temperature was reduced to 20/30 °C and the expression of SMO was induced by the addition of 0.25/0.5/1 mM of IPTG. To determine OD_600_ and level of SMO expression performed by denaturing polyacrylamide gel (SDS-PAGE) electrophoresis (Mini PROTEAN^®^ Tetra Cell, Bio-Rad, Hercules, CA, USA), 1 mL samples were taken regularly after induction, which took 22 h. The cells were then harvested by centrifugation (16,639× *g*, 6 °C, 30 min) and applied for biotransformation.

### 3.4. High Cell Density Fermentation

Fermentations were performed in 1.3/3 L New Brunswick BioFlo Bioreactors (Eppendorf, Hamburg, Germany) with an initial fermentation medium volume of 500 mL/1.5 L.

Preinoculum was prepared in a glass tube as described above. The inoculum was prepared by inoculation of 100 mL of M9 medium in a 500 mL shaking flask with 1% (*v*/*v*) of the preinoculum culture and cultivation at 37 °C, 200 rpm until OD_600_ reached 2.5. Fermentation was started by the inoculation of a bioreactor containing the semidefined medium with 2% (*v*/*v*) of inoculum culture. Temperature and pH were set to 37 °C and 7, maintained by the addition of 26% (*v*/*v*) ammonia and 3.1 M phosphoric acid. Air saturation of fermentation medium was maintained at dissolved oxygen (DO) = 30% by aeration (1 vvm) and agitation speed cascade (200–1200 rpm). When agitation reached 1200 rpm, the temperature was reduced to 30 °C and the expression of SMO was induced by the addition of 0.25 mM IPTG. To determine OD_600_, glycerol and acetic acid concentration, and specific activity and level of SMO expression, samples of fermentation medium were withdrawn regularly. Samples were processed as described above, except the supernatant was transferred to a fresh tube, diluted, and analysed by high-performance liquid chromatography (HPLC). Biomass for SMO activity determination was sampled separately and kept at 4 °C until the activity assay. Fermentation was terminated after complete substrate utilisation. Cells were harvested by centrifugation (16,639× *g*, 6 °C, 30 min) and stored at −80 °C.

### 3.5. Activity Assay

Harvested cells were resuspended in 100 mM potassium phosphate buffer (pH 8) to OD_600_ = 10 and disrupted by high-pressure homogeniser (276 MPa, 4 °C, 2 cycles) (CF Range, Constant Systems Ltd., Daventry, UK). Crude cell extract (CE) was clarified from cell debris by centrifugation (16,639× *g*, 6 °C, 30 min). The reaction mixture (1 mL) containing CE, glucose (100 mM), GDH (0.1 mg/mL), and NAD^+^ (1 mM) was prepared into 4 mL screw-neck vials and tempered in a shaking incubator at 30 °C and 200 rpm for 3 min. Biotransformation was started by the addition of 10 µL of styrene from 150 mM stock in methanol. To obtain a styrene oxide linear concentration increase, samples were withdrawn regularly and analysed by gas chromatography (GC). Each biotransformation was performed in two parallel runs, and given values represent mean values. One unit (U) was defined as the amount of SMO that catalyses the formation of 1 µM of styrene oxide in 1 min. The specific SMO activity was calculated as U per g of dry cell weight (U/g_DCW_).

### 3.6. Purification of Recombinant SMO

CE of SMO (biomass diluted to OD_600_ = 50) were prepared as described above. CE was filtered by vacuum filtration (Fisherbrand 0.45 µm; Fisher Scientific, PA, USA) or microfiltration (0.4 µm TangenX^TM^ PRO PDn Casette; Repligen, MA, USA) performed on an ÄKTA flux filtration unit (GE Healthcare, Chicago, IL, USA). Recombinant SMO was purified by fast protein liquid chromatography (FPLC) performed on an ÄKTA purifier/ÄKTA pilot unit equipped with 5 mL His Trap FF (GE Healthcare, Chicago, IL, USA)/50 mL Ni-NTA (Merck, Darmstadt, Germany) affinity column according to [26]. In brief, the column was equilibrated with binding buffer (10 mM imidazole, 300 mM NaCl, 0.3 mM sodium phosphate buffer pH 5.8), then the CE was loaded while the continual addition of binding buffer was remained to elute unbound proteins. Next, elution buffer (B) (500 mM imidazole, 300 mM NaCl, 0.3 mM sodium phosphate buffer, pH 7.4) was loaded into the column by 3-step concentration increase (25, 80, 100% of B) to elute nonspecific bounded proteins (at 25%) and His-tagged SMO (at 80%). Then, 50 µL of each eluted fraction was sampled to determine the presence of SMO by SDS-PAGE electrophoresis (Figure S6). Pooled SMO was concentrated and desalted in Amicon^®^ ultracentrifuge membrane (15 mL, 10 kDa; Merck, Darmstadt, Germany) or ultrafiltration cassette (10 kDa, TangenX^TM^ PRO PDn Casette, Repligen, MA, USA). The concentration of purified SMO was measured by an Eppendorf µCuvette^®^ (Eppendorf, Hamburg, Germany). The molar extinction coefficient of SMO was determined using ProtParam tool.

#### pH Profile and Temperature Profile

To determine the optimal pH, purified SMO was employed in the biotransformation of styrene. Reaction mixtures (1 mL) containing glucose (100 mM), GDH (0.3 mg/mL), SMO (1 mg/mL), NAD^+^ (1 mM), and potassium phosphate buffer (100 mM; pH 6.1, 6.4, 7.8, 8.7) were prepared. Biotransformations were performed as described above. To determine the optimal temperature, the same biotransformations were performed in a water bath (T = 20, 25, 30, 35, 40, and 45 °C) instead of a shaking incubator.

The storage stability of CE of SMO was determined, when 1 mL aliquots (100 mM potassium phosphate buffer, pH 8; OD_600_ = 50) were stored at 4, −20, and −80 °C. Reaction mixtures contained glucose (100 mM), GDH (0.3 mg/mL), CE (OD_600_ = 25), NAD^+^ (1 mM), and potassium phosphate buffer (100 mM, pH 8) or glucose (100 mM) and cell suspension (OD_600_ = 25) in potassium phosphate buffer (100 mM, pH 8) were prepared. Biotransformations were performed as described previously.

### 3.7. Biotransformation of Alkenes

Small-scale (1 mL) biotransformations were performed in 4 mL screw-neck vials employing CE of SMO (OD_600_ = 50) as described above. Additional biotransformations of 5, 10, and 20 mM **1f**, **2a**, **3b**, **5d**, **5g**, **5h**, **5j** alkenes were performed and allowed to proceed for 2–5 h. Specific SMO activity was expressed in U/g_DCW_. One unit (U) was defined as the amount of SMO that catalyses the formation /depletion (for **3b**) of 1 µmol of epoxide/alkene in 1 min.

Upscale (75–300 mL, specified in Table 3) of biotransformations was performed in a 500 mL baffled Erlenmeyer flask employing whole-cell SMO. The reaction mixture containing a cell suspension (OD_600_ = 50; 100 mM potassium phosphate buffer, pH 8) and glucose (100 mM) was tempered for 5 min in a shaking incubator at 30 °C and 200 rpm, then 1 mL of **1f**, **2a**, **5d**, **5g**, **5j** from a 2 M stock solution in methanol was added to start the biotransformation. Biotransformations were allowed to proceed for 9 h. To determine the conversion of alkenes and ee of corresponding epoxides, 100 µL samples were withdrawn for GC analysis. The rest of the reaction mixture was used for isolation and purification of corresponding epoxides performed immediately after biotransformation termination. All small-scale and upscale biotransformations were performed in two parallel runs.

### 3.8. Analytics

#### 3.8.1. High-Performance Liquid Chromatography

To determine glycerol and acetic acid concentrations, samples of cell-free fermentation medium were diluted with deionised water into a glass vial and analysed by an Agilent Infinity 1220 LC System (Agilent Technologies, Santa Clara, CA, USA) equipped with a WATREX polymer IEX H^+^ column (250 mm × 8 mm × 8 µm), an Agilent Infinity 1260 RI detector (Agilent Technologies, Santa Clara, CA, USA), and 9 mM H_2_SO_4_ as a mobile phase under the following conditions: injection volume 20 µL, temperature 50 °C, and flow 0.8 mL/min.

#### 3.8.2. Gas Chromatography

During all biotransformations, 100 µL of the reaction mixture was sampled regularly and extracted to 200 µL of ethyl acetate containing 0.25 mM of *n*-hexadecane as an internal standard by 30 s vortexing. The organic phase, containing the substrate and product, was separated from the water phase by centrifugation (14,000*× g*, 1 min) and transferred to a glass vial. To determine the conversion of biotransformation, samples were analysed by an Agilent 6890N gas chromatograph (Agilent Technologies, Santa Clara, CA, USA) equipped with a DB5 capillary column (Agilent J&W, 30 m × 0.25 mm × 0.25 µm), a flame-ionisation detector (FID), and H_2_ as a carrier gas under the following conditions: injection volume 1 µL, split 50:1, temperature gradient 90 °C (3.5 min), 30 °C/min to 280 °C; H_2_ flow 1.2 mL/min. Temperature gradients were adjusted according to the molecular weight of alkenes during substrate specificity screening. Conversion of biotransformation was established according to (1).

(1) $$\mathrm{conversion}\;=\;\frac{\left(\mathrm{peak}\;\mathrm{area}\;\mathrm{of}\;\mathrm{epoxide}\right)}{\left(\mathrm{peak}\;\mathrm{area}\;\mathrm{of}\;\mathrm{alkene}\;+\;\mathrm{peak}\;\mathrm{area}\;\mathrm{of}\;\mathrm{epoxide}\right)}\;\times \;100\%$$

To determine the ee of the products, samples were analysed by an Agilent 7890N gas chromatograph (Agilent Technologies, Santa Clara, CA, USA) equipped with a CP-Chirasil-Dex CB capillary column (Agilent J&W, 25 m × 0.25 mm × 0.25 µm), FID detector, and H_2_ as a carrier gas under the following conditions: injection volume 1 µL; split 50:1; temperature gradient 100 °C (0 min), 2 °C/min to 120 °C (1 min), 30 °C/min to 280 °C (1 min); H_2_ flow 1.2 mL/min.

### 3.9. Isolation and Characterisation of Chiral Epoxides

#### 3.9.1. General Information

Flash column chromatography (FCC) was carried out with a Büchi system (Pump Manager C-615 and Fraction Collector C-660) using Normasil 60 silica gel (0.040–0.063 mm; VWR). Thin layer chromatography (TLC) analysis was carried out using TLC silica gel 60 F_254_ (aluminium sheets, Merck), and plates were visualised with UV light or by treatment with permanganate solution followed by heating. Optical rotations were measured with a JASCO P-2000 digital polarimeter with a Na-D lamp (10 cm cell length). Concentrations (*c*) are given in grams per 100 mL. NMR spectra were recorded with a Varian INOVA-300 spectrometer (^1^H, 299.95 MHz) in CDCl_3_ using tetramethylsilane as the internal standard. Data are presented as follows: chemical shift (in ppm), multiplicity (bs = broad singlet, t = triplet, dd = doublet of doublets, m = multiplet), integration and coupling constants (J/Hz). All solvents used were dried and distilled according to conventional methods.

#### 3.9.2. General Procedure for the Isolation of Chiral Epoxides. Isolation of (*S*)-4-Chlorostyrene Oxide

The crude biotransformation mixture (100 mL) was vigorously stirred with ethyl acetate (150 mL) for 20 min. Then, the slurry was filtered through a pad of Celite, the organic phase was separated, and an aqueous layer was extracted with ethyl acetate (2 × 30 mL). The combined organic layers were dried over anhydrous MgSO_4_, and the solvent was evaporated under reduced pressure (max 120 mbar in a 25 °C water bath or max 50 mbar in a 35 °C water bath for long-chain aliphatic epoxides). The crude product was purified by FCC using hexanes as the eluent to give (*S*)-4-chlorostyrene (60 mg, 34% of theoretical yield) oxide as a colourless liquid with spectroscopic data in good agreement with those reported in the literature [36,37].

(*S*)-4-Chorostyrene Oxide


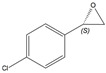


^1^H-NMR (300 MHz, CDCl_3_): δ = 2.75 (dd, 1H, *J* = 5.5, 2.5 Hz, OCH_2_), 3.14 (dd, 1H, *J* = 5.5, 4.1 Hz, OCH_2_), 3.83 (dd, 1H, *J* = 4.1, 2.5 Hz, OCH), 7.18–7.23 (m, 2H, H-Ar), 7.29–7.34 (m, 2H, H-Ar).

${\left[\alpha \right]}_{D}^{20}\;$= +27.3 (*c* 1.35, CHCl_3_).

*R*_f_ = 0.15 (*n-*hexane/ethyl acetate, 50:1).

Reported rotation for (*S*)-4-chlorostyrene oxide ${\left[\alpha \right]}_{D}^{20}$ = +26 (*c* 1.29, CHCl_3_) for 99% ee [36], ${\left[\alpha \right]}_{D}^{20}$ = +26 (*c* 1.2, CHCl_3_) for 99% ee [37].

(*S*)-Allylbenzene Oxide


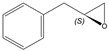


Yield: 26 mg (13% of theoretical yield), colourless liquid with spectroscopic data in good agreement with those reported in the literature [38].

^1^H-NMR (300 MHz, CDCl_3_): δ = 2.55 (dd, 1H, *J* = 5.0, 2.7 Hz, OCH_2_), 2.78–2.85 (m, 2H, PhCH_2_, OCH_2_), 2.93 (dd, 1H, *J* = 14.5, 5.6 Hz, PhC*H*_2_), 3.13–3.19 (m, 1H, OCH), 7.21–7.34 (m, 5H, H-Ar).

${\left[\alpha \right]}_{D}^{20}$= –17.1 (*c* 1.3, EtOH).

*R*_f_= 0.30 (*n*-hexane/ethyl acetate, 9:1).

Reported rotation for (*S*)-allylbenzene oxide ${\left[\alpha \right]}_{D}^{25}$ = −18.37 (*c* 2.01, EtOH) for 99% ee [39], ${\left[\alpha \right]}_{D}^{20}$ = −17.3 (*c* 1.0, EtOH) for 98% ee [40].

(2*R*,5*R*)-1,2:5,6-Diepoxyhexane


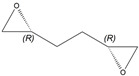


Yield: 157 mg (purified by FCC, hexanes/ethyl acetate, 9:1, 34% of theoretical yield), colourless liquid with spectroscopic data in good agreement with those reported in the literature [32].

^1^H-NMR (300 MHz, CDCl_3_): δ = 1.57–1.85 (m, 4H, CH_2_CH_2_), 2.51 (dd, 2H, *J* = 4.9, 2.7 Hz, OCH_2_), 2.78 (dd, 2H, *J* = 4.9, 4.0 Hz, OCH_2_), 2.91–3.01 (m, 2H, OCH).

${\left[\alpha \right]}_{D}^{26}$= +25.7 (*c* 1.2, CHCl_3_).

*R*_f_= 0.18 (*n-*hexane/ethyl acetate, 7:3).

Reported rotation for (2*R*,5*R*)-1,2:5,6-diepoxyhexane ${\left[\alpha \right]}_{D}^{26}$ = +26.8 (*c* 5.03, CHCl_3_) [41], ${\left[\alpha \right]}_{D}^{20}$ = +20.4 (*c* 1.3, CHCl_3_) [42].

*(S*)-4-(Oxiran-2-yl)butan-1-ol


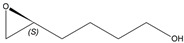


Yield: 76 mg (purified by FCC, hexanes/ethyl acetate, 4:1, 33% of theoretical yield), colourless liquid with spectroscopic data in good agreement with those reported in the literature [32].

^1^H-NMR (300 MHz, CDCl_3_): δ = 1.51–1.68 (m, 6H, CH_2_CH_2_CH_2_), 2.49 (dd, 1H, *J* = 5.0, 2.7 Hz, OCH_2_), 2.76 (dd, 1H, *J* = 5.0, 4.0 Hz, OCH_2_), 2.90–2.96 (m, 1H, OCH), 3.67 (t, 2H, *J* = 6.2 Hz, CH_2_OH).

${\left[\alpha \right]}_{D}^{25}$= −11.2 (*c* 1.0, CHCl_3_).

*R*_f_= 0.15 (*n-*hexane/ethyl acetate, 1:1).

Reported rotation for (*S*)-4-(oxiran-2-yl)butan-1-ol ${\left[\alpha \right]}_{D}^{24.7}$ = −12.8 (*c* 1.0, CHCl_3_) for >99.9% ee [32].

2-(3-Bromopropyl)oxirane


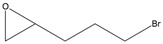


Yield: 173 mg (26% of theoretical yield) of the crude product (could not be purified by FCC because of its instability on silica gel), orange liquid with spectroscopic data in good agreement with those reported in the literature [43,44].

^1^H-NMR (300 MHz, CDCl_3_): δ = 1.53–1.66 (m, 1H, CH_2_CH_2_), 1.76–1.88 (m, 1H, CH_2_CH_2_), 1.98–2.08 (m, 2H, CH_2_CH_2_), 2.51 (dd, 1H, *J* = 4.9, 2.7 Hz, OCH_2_), 2.77 (dd, 1H, *J* = 4.9, 4.0 Hz, OCH_2_), 2.92–2.98 (m, 1H, OCH), 3.41–3.53 (m, 2H, C*H*_2_Br).

*R*_f_ = 0.19 (*n-*hexane/ethyl acetate, 7:3).

## 4. Conclusions

Herein, the production of recombinant SMO by HCD fermentation that yielded 35 g_DCW_/L was optimised. It was found that SMO loses stability in the isolation process and was not suitable for biotransformations in a purified form. The highest specific activity, stability, enantioselectivity, and purity of desired products was achieved by application of whole-cell SMO, which was proven to be the most suitable form for epoxidation of alkenes. In its crude form, SMO exhibited the highest activity at pH 8 and 35 °C and retained 70% of its initial activity within 16 months of storage. The enzyme transformed 7 out of 34 tested alkenes to corresponding epoxides, many of them with higher specific activity than styrene. Eventually, (*S*)-4-chlorostyrene oxide, (*S*)-allylbenzene oxide, (2*R*,5*R*)-1,2:5,6-diepoxyhexane, 2-(3-bromopropyl)oxirane, and (*S*)-4-(oxiran-2-yl)butan-1-ol were produced with excellent enantiopurity (ee > 99%, 95%, 97%, 99%, and 99%, respectively).

## Figures and Tables

**Table 1:** Results of HCD fermentations.

Final Volume (L)	Cell Concentration (g_DCW_/L)	Total Dry Cell Weight (g_DCW_)	Enzyme Activity (U/g_DCW_)	Total Activity (U)
0.5 L	31	15.5	10.5	162.8
1.5 L	35	52.5	9.6	504

**Table 2:** Substrate specificity of recombinant CE of SMO.

Structure	Substituents	Entry	Activity (U/g_DCW_)	Conversion (%)
** 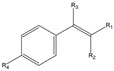 **	R_1_, R_2_, R_3_, R_4_ = H	**1a**	7	99
	R_1_, R_2_, R_4_ = H; R_3_ = Br	**1b**	-	-
	R_1_ = CHO, R_2_, R_3_, R_4_ = H	**1c**	-	-
	R_1_ = CHO, R_2_, R_3_ = H, R_4_ = OCH_3_	**1d**	-	-
	R_1_ = CHO, R_2_, R_3_ = H, R_4_ = NO_2_	**1e**	-	-
	R_1_, R_2_, R_3_ = H, R_4_ = Cl	**1f**	12	90
** 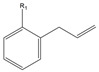 **	R_1_ = H	**2a**	23	87
	R_1_ = OH	**2b**	-	-
** 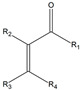 **	R_1_, R_2_, R_3_, R_4_ = H	**3a**	-	-
	R_1_, R_3_, R_4_ = H; R_2_ = CH_3_	**3b**	13	100
	R_1_ = CH_3_; R_2_, R_3_, R_4_ = H	**3c**	-	-
	R_1_, R_2_, R_3_ = H; R_4_ = (CH_2_)_2_CH_3_	**3d**	-	-
	R_1_ = Cl; R_2_, R_3_, R_4_ = H	**3e**	-	-
** 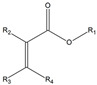 **	R_1_ = OH; R_2_, R_3_, R_4_ = H	**4a**	-	-
	R_1_ = CH_3_; R_2_, R_3_, R_4_ = H	**4b**	-	-
	R_1_, R_2_ = CH_3_; R_3_, R_4_ = H	**4c**	-	-
	R_1_, R_3_ = CH_3_; R_2_, R_4_ = H	**4d**	-	-
	R_1_ = (CH_2_)_3_CH_3_; R_2_, R_3_, R_4_ = H	**4e**	-	-
	R_1_ = CH_2_CH_3_; R_2_, R_3_ = H; R_4_ = CHO	**4f**	-	-
** 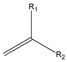 **	R_1_ = H, R_2_ = CH_2_OH	**5a**	-	-
	R_1_ = CH_3_, R_2_ = CH_2_OH	**5b**	-	-
	R_1_ = H, R_2_ = (CH_2_)_3_OH	**5c**	-	-
	R_1_ = H, R_2_ = (CH_2_)_4_OH	**5d**	25	99
	R_1_ = H, R_2_ = CH(OH)(CH_2_)_3_CH_3_	**5e**	-	-
	R_1_ = H, R_2_ = CH(OH)(CH_2_)_2_	**5f**	-	-
	$R_{1}=H,\;R_{2}=({\mathrm{CH}}_{2}{)}_{2}\mathrm{CH}(O)\mathrm{CH}2_{\;}$ 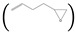	**5g**	5	99
	$R_{1}=H,\;R_{2}=({\mathrm{CH}}_{2}{)}_{4}\mathrm{CH}(O)\mathrm{CH}2_{\;}$ 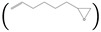	**5h**	3	67
	R_1_ = Br, R_2_ = CH_3_	**5i**	-	-
	R_1_ = H, R_2_ = (CH_2_)_3_Br	**5j**	7	99
	R_1_ = H, R_2_ = CN	**5k**	-	-
	R_1_ = H, R_2_ = CH(OCH_2_CH_3_)_2_	**5l**	-	-
** 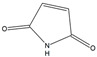 **		**6**	-	-
** 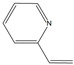 **		**7**	-	-
** 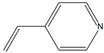 **		**8**	-	-

**Table 3:** Summary of upscaled biotransformations by whole-cell SMO.

Entry	Substrate	Product	Configuration	ee (%)	Conversion (%)	Yield (mg)	Reaction Volume/Yield (mL/mg)
**1f**	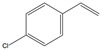	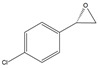	*S*	>99%	99	60	1.27
**2a**	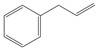	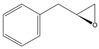	*S*	>95%	99	26	2.88
**5g**	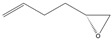	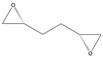	2-*R*,5-*R*	>97%	99	157	1.28
**5j**	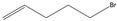	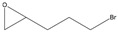	*ND* ^1^	>99% ^2^	93	173	1.73
**5d**	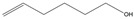	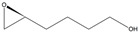	*S*	>99% ^3^	99	76	1.32

^1^ Not determined due to low stability of epoxide. ^2^ Determined by chiral GC-FID. ^3^ Based on optical rotation value.

## Data Availability

The data presented in this study are available in Supplementary Materials.

## Supplementary Materials

The following are available online, Figure S1: Cell growth after induction of SMO expression (0.25 mM IPTG) in LB and M9 medium and final specific activity of SMO, Figure S2: SDS-PAGE electrophoresis of induction of SMO expression by different concentration of IPTG. OD_600_ = 0.7–0.8. Lane 1–protein ladder; Lane 2, 3–0.25 mM IPTG 0, 4 h; Lane 4, 5–0.5 mM IPTG 0, 4 h; Lanes 6, 7, 8, 9–1 mM IPTG 0, 2, 3, 4 h; Lane 10—cell pellet (inclusion bodies); Lane 11–cell extract (soluble SMO); Lane 12—protein ladder, Figure S3: SDS-PAGE electrophoresis of induction of SMO expression at 20 and 30 °C in LB and M9 medium. OD_600_ = 0.4–0.5. Lanes 1, 2, 3—LB, 20 °C whole cells, crude extract, pellet; Lanes 4, 5, 6—LB, 30 °C whole cells, crude extract, pellet; Lane 7–protein ladder, Lanes 8, 9, 10—M9, 20 °C whole cells, crude extract, pellet; Lanes 11, 12, 13—M9, 30 °C whole cells, crude extract, pellet, Figure S4: HCD batch fermentation of *E. coli* expressing SMO performed on the 0.5 L scale, Figure S5: Protein profile of *E. coli* after induction of SMO expression during HCD fermentation. Lane 1: protein ladder, Lane 2: 0 h, Lane 3: 2 h, Lane 4: 4 h, Lane 5: 6 h, Lane 6: 7.5 h after induction. Figure S6: Isolation of SMO by immobilised Ni^2+^ affinity chromatography, Figure S7: Temperature profile of SMO in form of crude extract, Figure S8: Storage of SMO in form of whole cells (a) and crude extract (b), Figure S9: Repeated biotransformation of styrene by whole-cell SMO, Scheme S1. Retrosynthesis of (*R*)-4-chlorostyrene oxide (**a**) [37], (*R*)-3-chlorostyrene oxide (**b**) [35], and 2-benzyl-2-methyloxirane (**c**) [35], Figure S10: ^1^H-NMR spectrum of (*S*)-4-chlorostyrene oxide, Figure S11: ^1^H-NMR spectrum of (*S*)-allylbenzene oxide, Figure S12: ^1^H-NMR spectrum of (2*R*,5*R*)-1,2:5,6-diepoxyhexane, Figure S13: ^1^H-NMR spectrum of (*S*)-4-(oxiran-2-yl)butan-1-ol, Figure S14: ^1^H-NMR spectrum of 2-(3-bromopropyl)oxirane, Table S1: Summarised results of SMO purification, Table S2. The evaluation of SMO specific activity during purification.

## Sample Availability

Samples of enzymes and epoxides are available, contact corresponding author.
