# Supplementary material for: Production of Enantiopure Chiral Epoxides with E. coli Expressing Styrene Monooxygenase
Source: Molecules. 2021 Mar 10;26(6):1514. doi: 10.3390/molecules26061514 (PMC8001364; doi:10.3390/molecules26061514)
Supplement: Supplementary file 1 [file molecules-26-01514-s001.pdf]

molecules

Supplementary Materials: Production of Enantiopure Chiral Epoxides with *E. coli* Expressing Styrene Monooxygenase

Dominika Gyuranová [1], Radka Štadániová [2], Zuzana Hegyi [1], Róbert Fischer [2] and Martin Rebroš [1,*]

[1] Institute of Biotechnology, Faculty of Chemical and Food Technology, Slovak University of Technology in Bratislava, Radlinského 9, 812 37 Bratislava, Slovakia; dominika.gyuranova@stuba.sk; zuzana.hegyi@stuba.sk
[2] Institute of Organic Chemistry, Catalysis and Petrochemistry, Faculty of Chemical and Food Technology, Slovak University of Technology in Bratislava, Radlinského 9, 812 37 Bratislava, Slovakia; radka.stadaniova@stuba.sk; robert.fischer@stuba.sk
* Correspondence: martin.rebros@stuba.sk; Tel.: +421-2-59-325-480

1. Expression of SMO

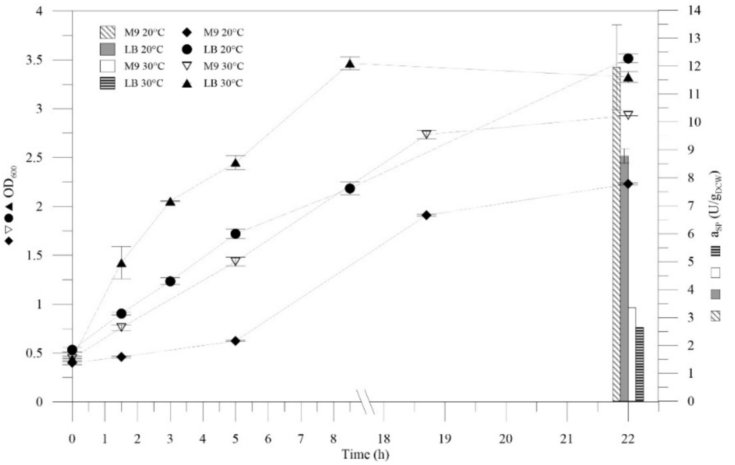

Figure S1. Cell growth after induction of SMO expression (0.25 mM IPTG) in LB and M9 medium and final specific activity of SMO.

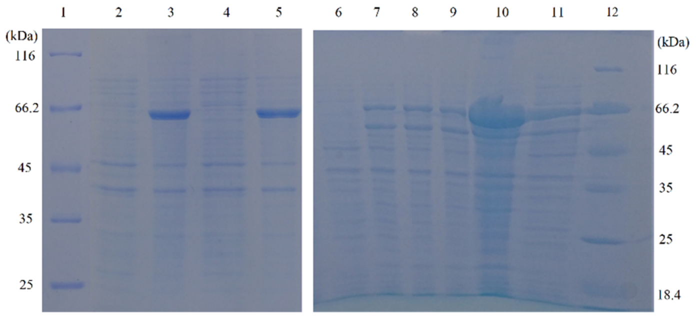

Figure S2. SDS-PAGE electrophoresis of induction of SMO expression by different concentration of IPTG. OD_{600} = 0.7 – 0.8. Lane 1 – protein ladder; Lane 2, 3 – 0.25 mM IPTG 0, 4 h; Lane 4, 5 – 0.5 mM IPTG 0, 4 h; Lane 6, 7, 8, 9 – 1 mM IPTG 0, 2, 3, 4 h; Lane 10 – cell pellet (inclusion bodies); Lane 11 – cell extract (soluble SMO); Lane 12 – protein ladder.

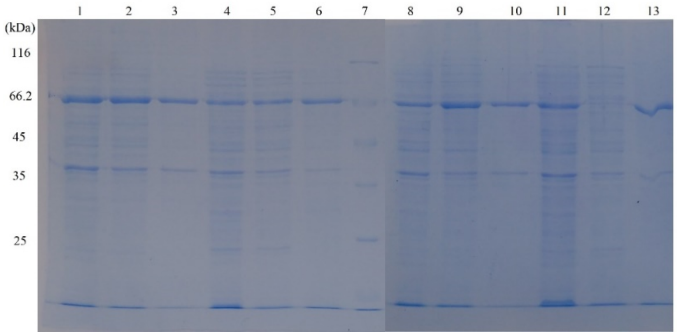

Figure S3. SDS-PAGE electrophoresis of induction of SMO expression at 20 and 30 °C in LB and M9 medium. OD_{600} = 0.4 – 0.5. Lane 1, 2, 3 – LB, 20 °C whole cells, crude extract, pellet; Lane 4, 5, 6 – LB, 30 °C whole cells, crude extract, pellet; Lane 7 – protein ladder, Lane 8, 9, 10 – M9, 20 °C whole cells, crude extract, pellet; Lane 11, 12, 13 – M9, 30 °C whole cells, crude extract, pellet.

2. High cell density fermentation

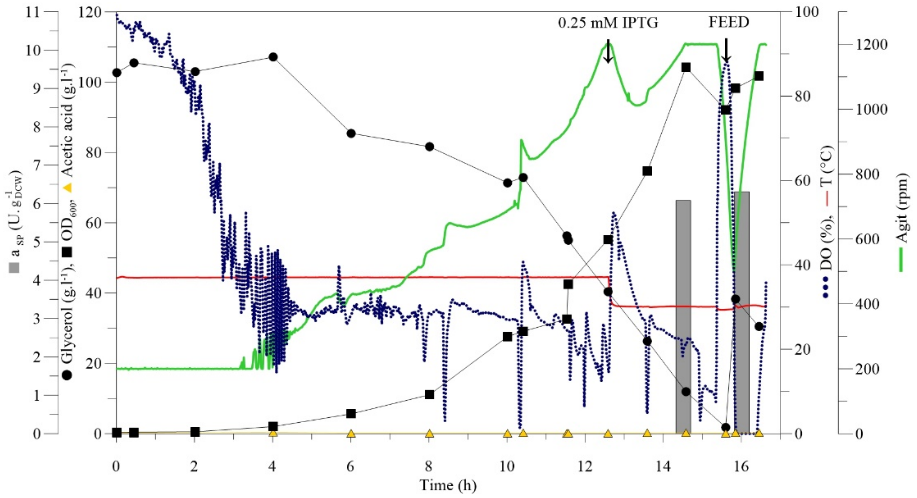

Figure S4. HCD batch fermentation of *E. coli* expressing SMO performed on the 0.5 L scale.

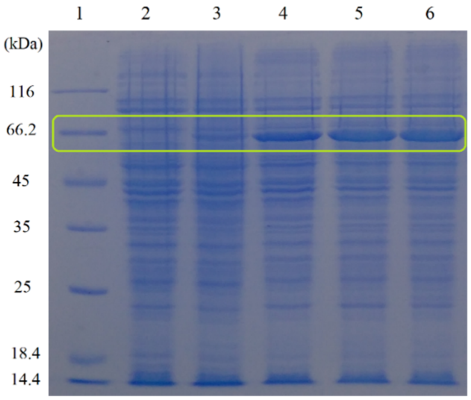

Figure S5. Protein profile of *E. coli* after induction of SMO expression during HCD fermentation. Lane 1: protein ladder, Lane 2: 0 h, Lane 3: 2 h, Lane 4: 4 h, Lane 5: 6 h, Lane 6: 7.5 h after induction.

3. Purification of recombinant SMO

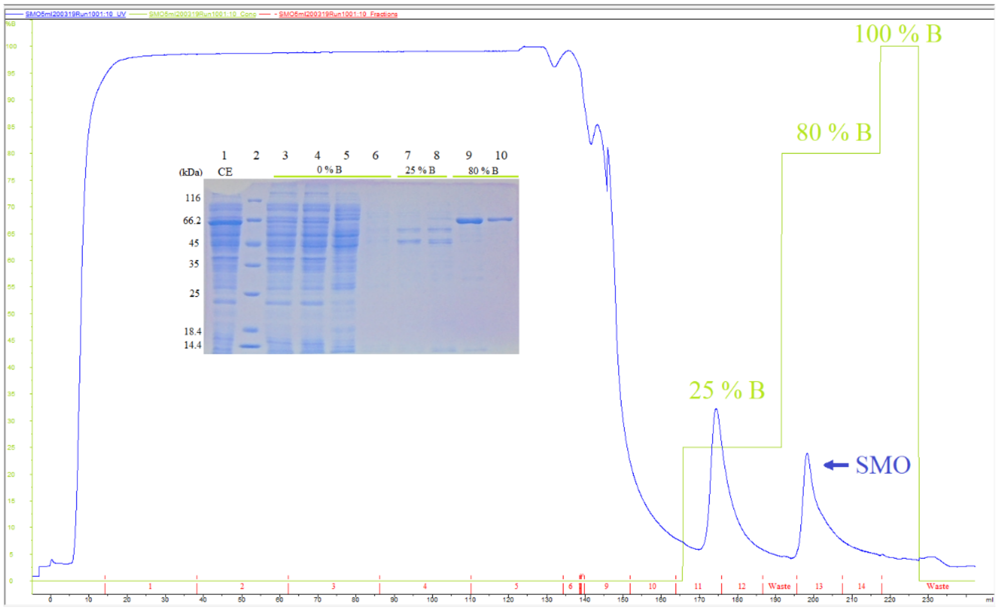

Figure S6. Isolation of SMO by immobilised Ni^{2+} affinity chromatography.

Table S1. Summarised results of SMO purification.

Resin volume (ml)	Loaded crude extract volume (ml)	Recovered SMO amount (mg)	Yield (mg/mL$_{CE}$)
5	50	8.4	0.2
50	300	34	0.1

4. Biotransformation of alkenes

Table S2. The evaluation of SMO specific activity during purification.

The form of SMO	Specific SMO activity (U/g$_{DCW}$)
whole-cell	9.3 ± 0.5
CE	8.1 ± 0.4
purified	0.03 ± 0.003

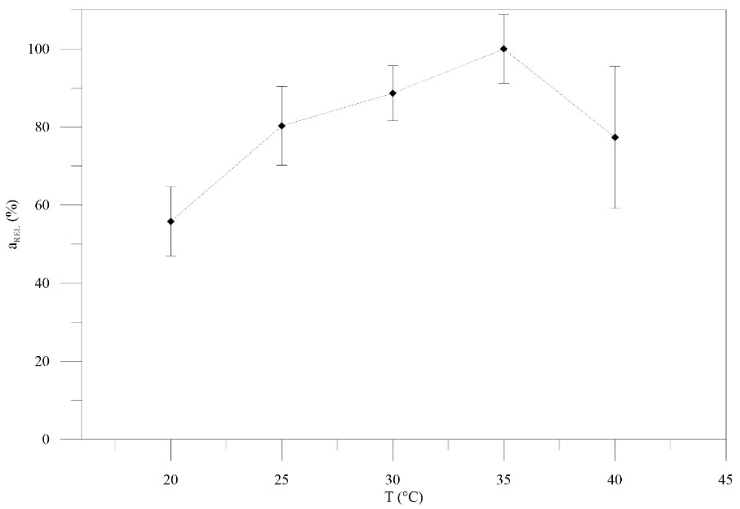

Figure S7. Temperature profile of SMO in form of crude extract.

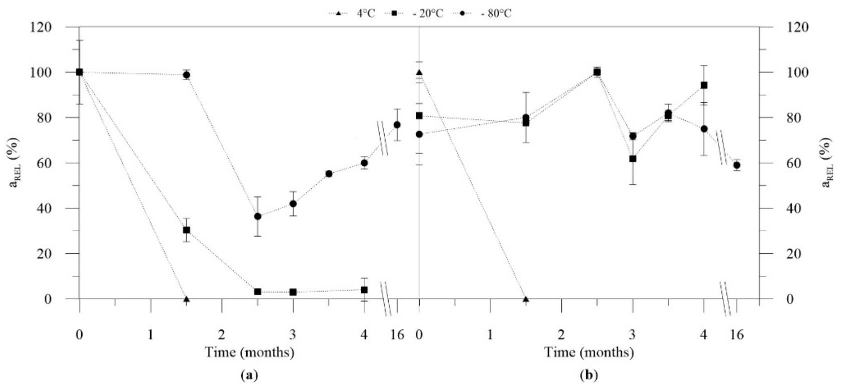

Figure S8. Storage of SMO in form of whole cells (**a**) and crude extract (**b**).

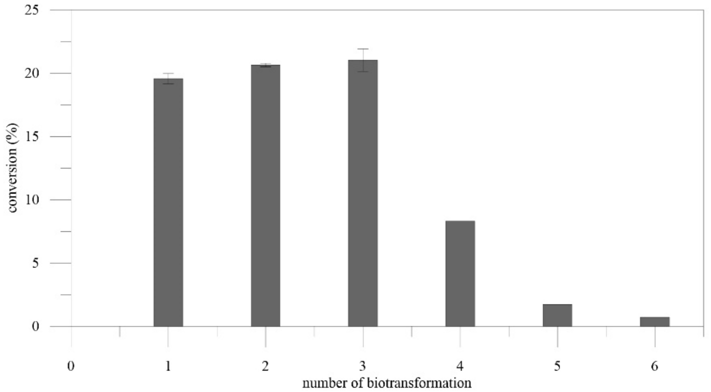

Figure S9. Repeated biotransformation of styrene by whole-cell SMO.

5. Upscale production of chiral epoxides

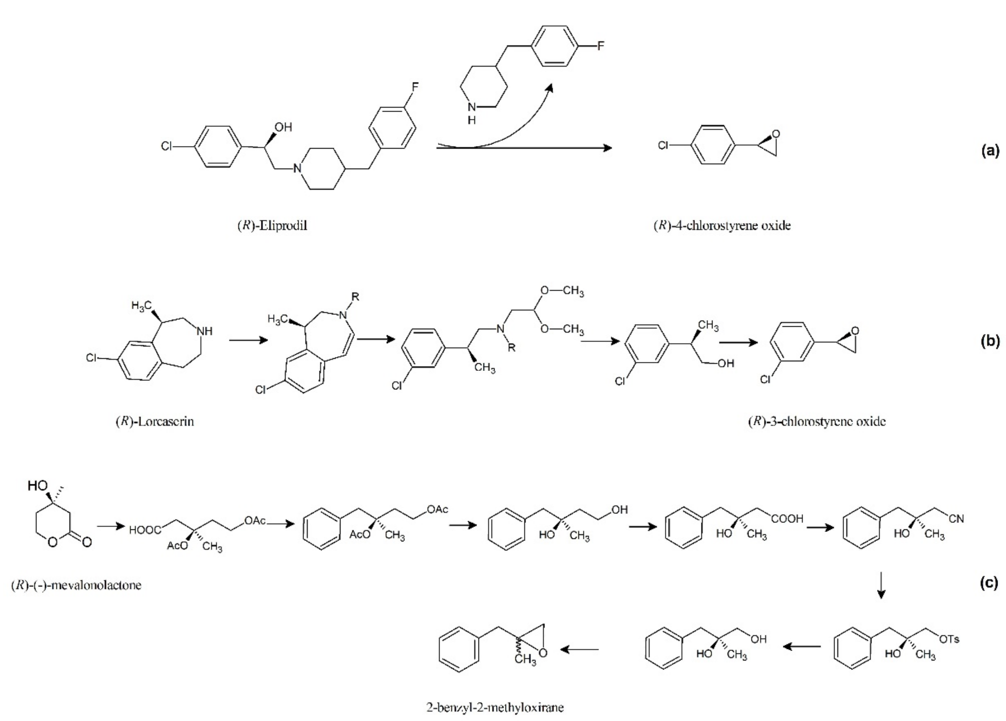

Scheme S1. Retrosynthesis of (*R*)-4-chlorostyrene oxide **(a)** [37], (*R*)-3-chlorostyrene oxide **(b)** [35], and 2-benzyl-2-methyloxirane **(c)** [35].

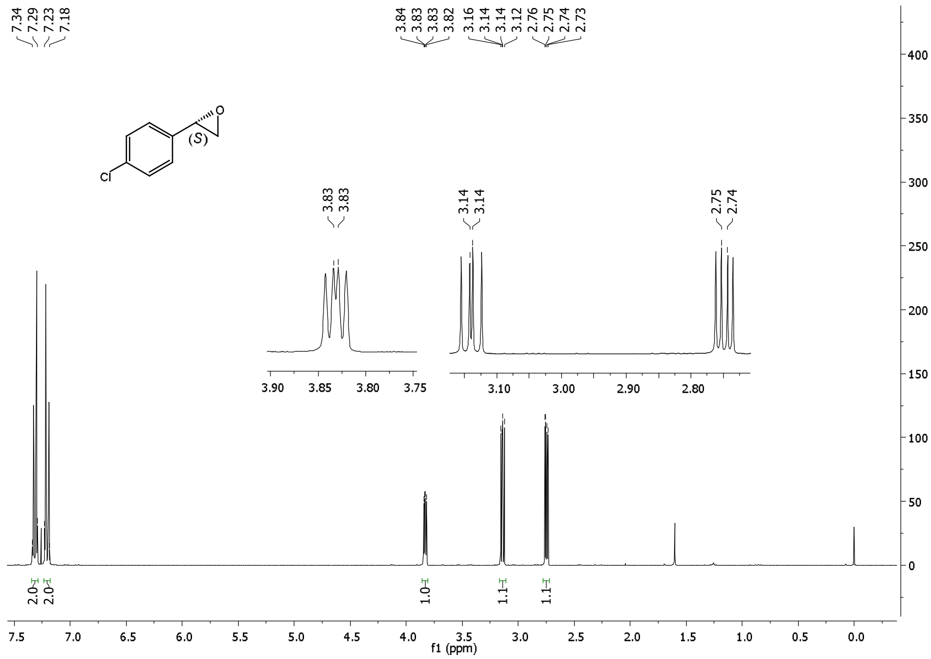

Figure S10. ^{1}H NMR spectrum of (*S*)-4-chlorostyrene oxide.

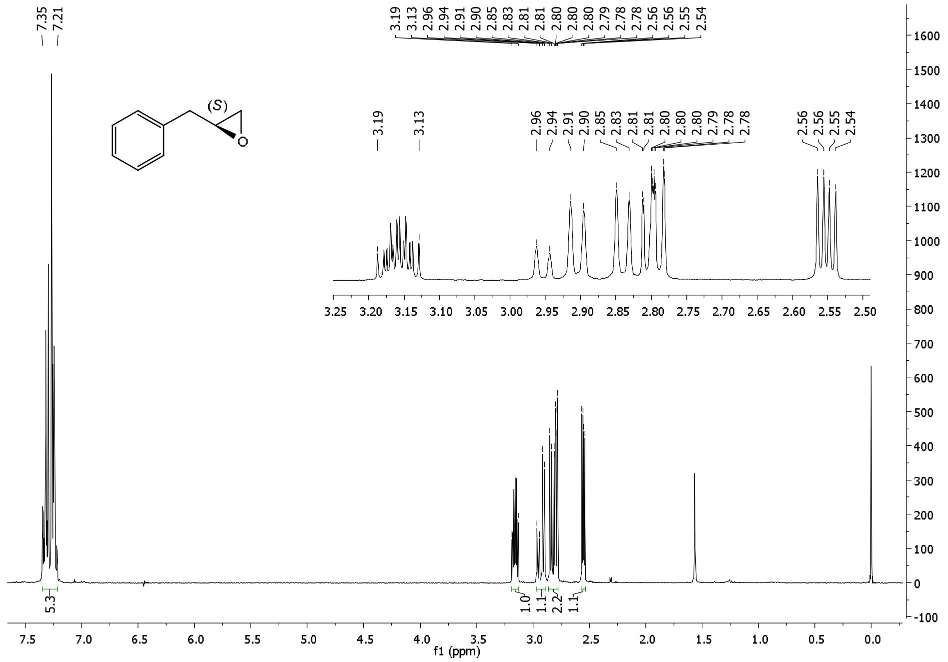

Figure S11. ^{1}H NMR spectrum of (*S*)-allylbenzene oxide.

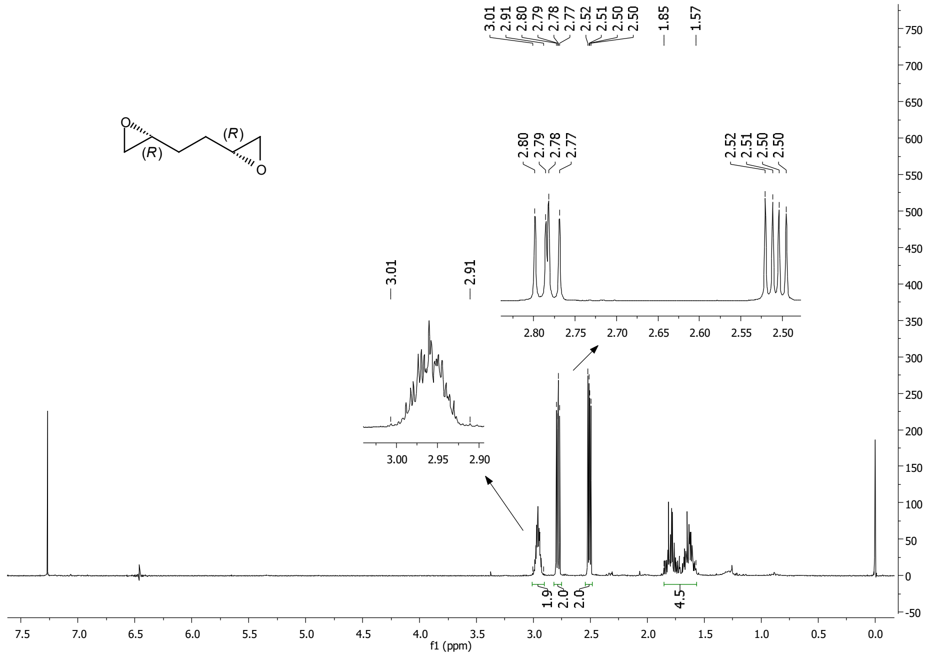

Figure S12. ^{1}H NMR spectrum of (2*R*,5*R*)-1,2:5,6-diepoxyhexane.

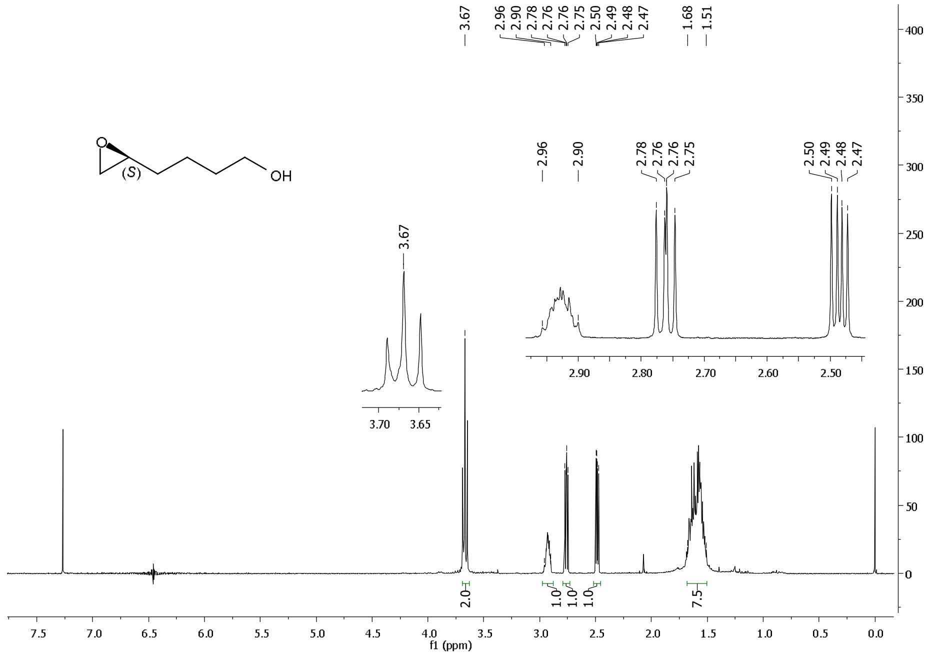

Figure S13. ¹H NMR spectrum of (*S*)-4-(oxiran-2-yl)butan-1-ol.

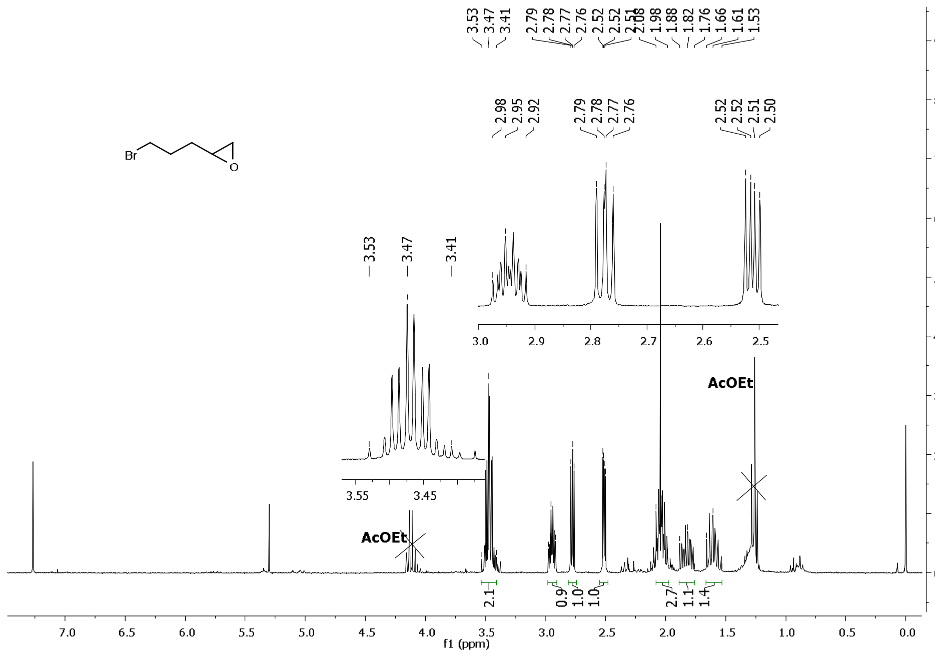

Figure S14. ¹H NMR spectrum of 2-(3-bromopropyl)oxirane.
